# Therapeutic Pearls at Random Strung

**Published:** 1881-07

**Authors:** 


### THERAPEUTIC PEARLS AT RANDOM STRUNG.

### Leucorrhœa.

No. 1.

℞ Hydrarg. Bichlor., . . . gr. i.
Tinct. Aconite, Fol.
" Cantharides, . . . āā ℨ ss.
Morph. Sulph. . . . . . grs. i.
Syrup Simp. vel. Acæ . . . ℥ ij. M.

S. A teaspoonful ter die, and use injections of Sulph. Zinc, Alum, Tannin, &c.

The following recipe with two to five grains of Cornina, (active principle of Cornus Florida) every three or four hours, is regarded as a specific by some in *uterine* leucorrhœa, with or without recipe No. 1, which is so beneficial in *vaginal* leucorrhœa.

No. 2.

℞ Ol. Erigeron, . . . . .

Ol. Stillingia, Sil. . . . āā ℨ ij. M.

S. Two drops ter die.

### Neuralgia.

Ovarian Neuralgia is often promptly relieved by the following recipe:

℞ Ammonia Muriat., . . . ℨ ij.

Tinct. Aconite, . . . . f. ℨ ij.

Syrup Aurantii Cor., . . . f. ℥ viij. M.

Teaspoonful three times a day.

### Neuralgic Formula of Dr. E. Brown-Sequard.

℞ Ext. Belladon. . . . gr. 1-6
" Stramon. . . . . gr. 1-5
" Can. Indica. . . . . gr. 1-4
" Aconit. . . . . gr. 1-3
" Opii. . . . . gr. ½
" Hyosciami. . . . . gr. 2-3
" Cornii. . . . . gr. 1
Pulv. Glycyr. . . . . . q. s.
For one pill.

S. Three to five pills a day.

GASTRALGIA.

Dr. Edward Vanderpool, of New York, recommends Fowler's Solution of Arsenic in neuralgia of the stomach, in six to ten drops three times per day. His experience with it appears to have been highly satisfactory in the cases reported.

NEURALGIA, OR RHEUMATISM.

℞ Chloroform, Tinct. Aconite Rad. . . āā f. ʒ ij

Morphia Sulph. . . . . . . gr. i.

Iodide Potass. . . . . . . ʒ i. M.

Prick the skin with a fine needle over the seat of pain with twenty or thirty punctures, and rub in the foregoing mixture. Immediate relief is said to follow each application, and a cure effected in local cases in a short time.

PEPPERMINT IN NEURALGIA AND RHEUMATISM.

The Chinese apply peppermint locally over the seat of pain with a camel's hair pencil. It has afforded immediate relief also in gout and rheumatism, when thus applied.

Dr. George W. Laurence, of Hot Springs, Ark., recommends the following recipe in all cases requiring local or revulsive action. Dilute the mixture in ulcerated surfaces and skin diseases:

℞ Carbolic acid, (Calvert's) . . . . ʒ i.
Acetic Acid f. (pure) . . . . . ℥ ss.
Water, . . . . . . f. ℥ ss. M.

S. Apply with a camel's hair brush, once or twice a day, in neuralgia, paralysis, rheumatism, Locomoter ataxy, etc., etc. It is said to be more prompt and efficacious than mustard and kindred applications.

